# Sand fly fauna of South-Eastern Romania, with the description of *Phlebotomus* (*Transphlebotomus*) *simonahalepae* n. sp. (Diptera: Psychodidae)

**DOI:** 10.1186/s13071-021-04929-6

**Published:** 2021-09-06

**Authors:** Cristina Daniela Cazan, Attila D. Sándor, Ozge Erisoz Kasap, Bulent Alten, Andrei Daniel Mihalca

**Affiliations:** 1 CDS-9: Molecular Biology and Veterinary Parasitology Unit, Faculty of Veterinary Medicine, University of Agricultural Sciences and Veterinary Medicine, Cluj-Napoca, Romania; 2 Department of Parasitology and Parasitic Diseases, Faculty of Veterinary Medicine, University of Agricultural Sciences and Veterinary Medicine, Cluj-Napoca, Romania; 3 Department of Parasitology and Zoology, University of Veterinary Medicine, Budapest, Hungary; 4 Department of Biology, Ecology Section, Faculty of Science, VERG Laboratories, Hacettepe University, Ankara, Turkey

**Keywords:** Sand flies, *Phlebotomus neglectus*, *Phlebotomus simonahalepae* n. sp., *Transphlebotomus*, Romania

## Abstract

**Background:**

An entomological study was conducted in the Canaraua Fetii Special Protection Area in the Dobrogea region, South-Eastern Romania. Four sand fly species were recorded at this location between 1968 and 1970: *Phlebotomus neglectus*, *Ph*. *balcanicus*, *Ph*. *sergenti* and *Sergentomyia minuta*. The most abundant sand fly species recorded at that time were *Ph*. *balcanicus* and *Se*. *minuta*. In the context of a countrywide study to update the sand fly species diversity, we surveyed the same area, recording also a previously unknown *Ph*. (*Transphlebotomus*) sp., for which we provide a formal description here.

**Methods:**

Sand flies were collected between July and August in 2018 and 2019 in three sites from Canaraua Fetii, Dobrogea region, Romania. The general aspect of the landscape is of a canyon (vertical, narrow walls and deep valleys). Species identification was done using both morphological and molecular analyses.

**Results:**

Out of 645 collected sand flies, 644 (99.8%) were morphologically identified as *Ph*. *neglectus*, while one female specimen (0.2%) was assigned to a previously unknown species, belonging to the subgenus *Transphlebotomus*. The morphological and molecular examination of this specimen showed that it is a previously unknown species which we formally describe here as *Phlebotomus* (*Transphlebotomus*) *simonahalepae* n. sp. Also, *Ph*. *balcanicus*, *Ph*. *sergenti*, and *Se*. *minuta* (previously recorded in this location) were not present.

**Conclusions:**

The study revealed for the first time the presence of sand flies of the subgenus *Transphlebotomus* in Romania. Moreover, a new species, *Ph*. *simonahalepae* n. sp., was described based on a female specimen, raising the number of species in this subgenus to six. In the investigated natural habitat, the predominant species was *Ph*. *neglectus* instead of *Ph*. *balcanicus* and *Se*. *minuta* (recorded as the predominant species in 1968–1970).

**Graphical abstract:**

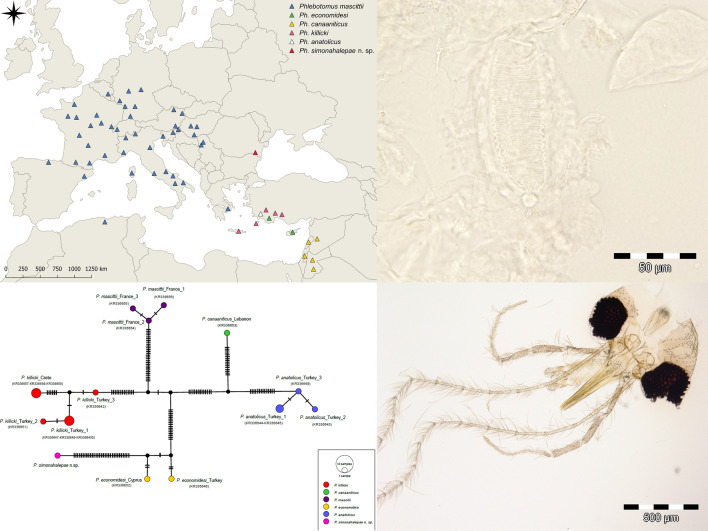

**Supplementary Information:**

The online version contains supplementary material available at 10.1186/s13071-021-04929-6.

## Background

Phlebotomine sand flies (Diptera, Psychodidae, Phlebotominae) are important hematophagous insects of public health concern in both the Old and New World [1]. Sand flies play a major role in the transmission of the parasites of genus *Leishmania* (Kinetoplastida, Trypanosomatidae), but also bacterial and viral pathogens [2].

In Europe, sand flies are mostly present in the Mediterranean basin, highly endemic for zoonotic visceral leishmaniasis in humans (VL) and canine leishmaniasis (CanL) in dogs, caused by *Leishmania infantum* [2]. Three species that are vectors for *L*. *infantum* are present in Romania: *Phlebotomus perfiliewi*, *Ph*. *neglectus*, and *Ph*. *balcanicus* [2, 3]. In recent years, sporadic autochthonous cases of both VL and CanL have been reported at the northern limit of sand fly distribution, including Romania [4]. The permanent risk of VL and CanL emergence in new areas requires constant surveillance of vector presence and abundance and disease epidemiology, mainly at the limit of their distribution [4].

Eight sand fly species were recorded in Romania between 1910 and 1970: *Ph*. (*Larroussius*) *perfiliewi* Parrot, 1930; *Ph*. (*Larroussius*) *neglectus* Tonnoir, 1921; *Ph*. (*Adlerius*) *balcanicus* Theodor, 1948; *Ph*. (*Phlebotomus*) *papatasi* (Scopoli, 1786); *Ph*. (*Paraphlebotomus*) *alexandri* Sinton, 1928; *Ph*. (*Paraphlebotomus*) *sergenti* Parrot, 1917; *Ph*. (*Adlerius*) *longiductus* Parrot, 1928; and *Sergentomyia* (*Sergentomyia*) *minuta* (Rondani, 1843) [5]. The highest sand fly diversity recorded between 1968 and 1970 was found in the protected natural habitat of Canaraua Fetii, Dobrogea region, South-Eastern Romania, with four sand fly species: *Ph*. *neglectus*, *Ph*. *balcanicus*, *Ph*. *sergenti*, and *Se*. *minuta* [6].

In a more recent study conducted between 2013 and 2018, only five sand fly species were identified in Romania: *Ph*. *perfiliewi*, *Ph*. *neglectus*, *Ph*. *balcanicus*, *Ph*. *papatasi*, and *Ph*. *sergenti* [3]. Currently, the Mehedinţi Plateau (South-Western Romania) is the region with the highest sand fly species diversity described in Romania, with five species recorded [3]. Three other species recorded as present in Romania between 1910 and 1970, *Ph*. *alexandri*, *Ph*. *longiductus*, and *Se*. *minuta*, were not identified in recent surveys [3].

Herein, we describe a previously unknown *Phlebotomus* (*Transphlebotomus*) sp. which has been found during a countrywide study to update the sand fly species diversity in Romania.

## Methods

### Study area and design

**Fig. 1:**
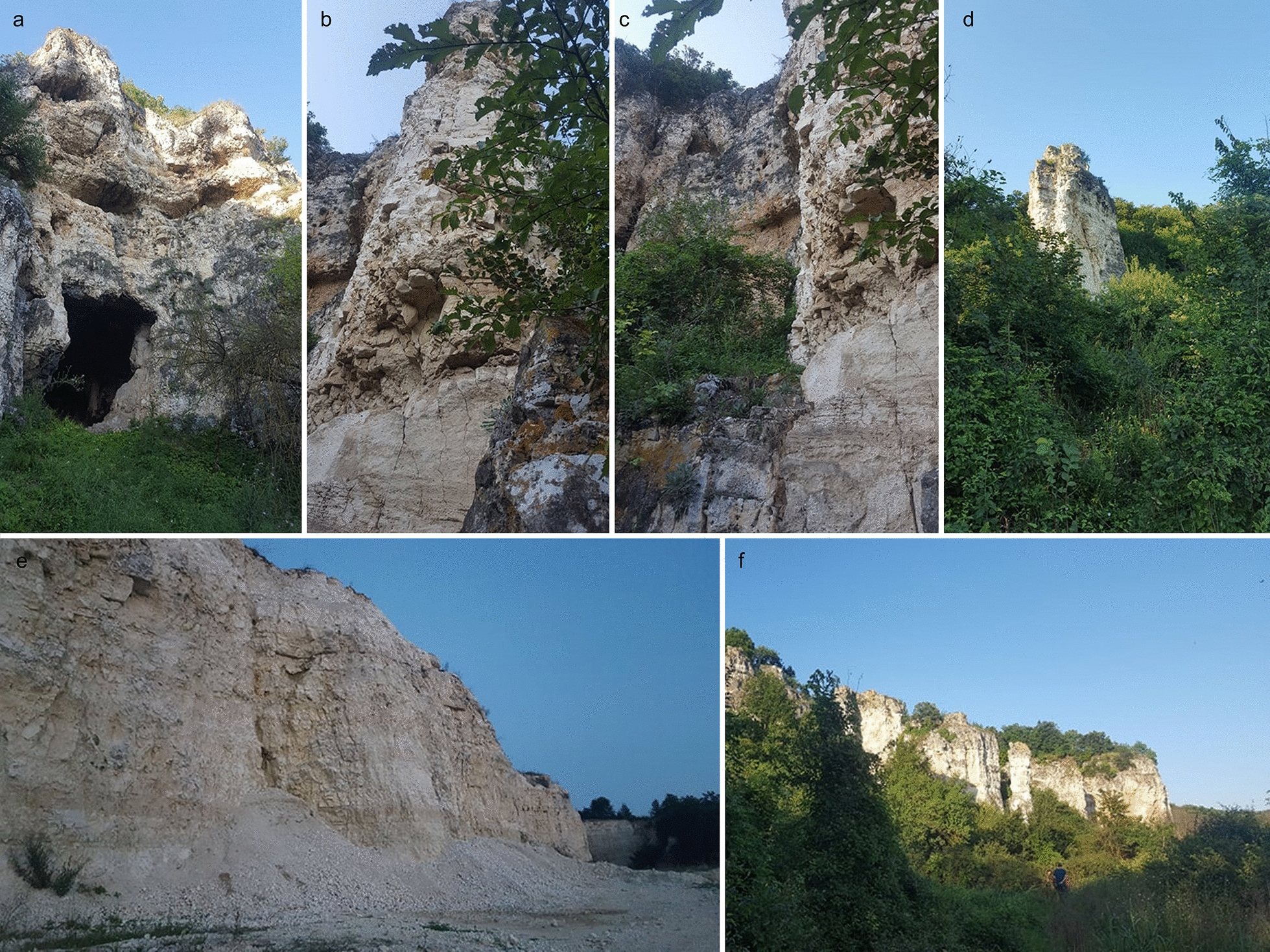
Canaraua Fetii, Dobrogea Region, Romania. **a** Cave entrance. **b**, **c**, **d** Limestone formations. **e** The specific collection site for the current study. **f** General view of the natural reserve

Between 31 July and 2 August 2018 and 29 July and 1 August 2019, CDC light traps (John W. Hock Company, USA) and sticky traps were placed in the protected area of Canaraua Fetii in South-Eastern Romania (44.07302 N, 27.64289 E). Mouth aspirators were also used to collect sand flies directly from the walls of caves and crevices or while biting the researchers. The protected area is situated in south-western part of Dobrogea Plateau. It is a limestone canyon (Fig. 1), carved by a former river among hills forming a plateau. It has deciduous forests on the sides and typical short-grass steppes on top. The valley is moist (a temporary brook crosses, with slow-flowing water following rains), while the plateau is drier. Elevation is 100–130 m on the plateau, 18–26 m in the valley. The area holds a high diversity of animal species, with important bird and bat populations noted [7]. Six CDC light traps were set in three sites, for three consecutive nights in 2018 and for four consecutive nights in 2019, in order to assess the presence/absence of the sand fly species. A standardized protocol was used [8].

The trapping sites were represented by two cave entrances and a former abandoned, windowless construction, all these being used by diverse bat populations. The total number of light traps/days placed in the study was 42 [2 traps × 3 premises × 3 consecutive nights (2018) × 1 time (2018) + 2 traps × 3 premises × 4 consecutive nights (2019) × 1 time (2019)]. The CDC light traps were set overnight (19:00–05:30) near the walls, at a height of 1.5 m from the ground. In 2019, the light trap collections were complemented with the use of sticky traps. Sticky traps consisted of A5 format white paper (148 mm × 210 mm) coated with castor oil; a fixed number of sticky traps per site (*n* = 10) were set in each trapping site during the sampling period.

### Species identification

After each trapping night, insects were collected, stored in 70% ethanol, and transferred to the laboratory for species identification. Sand flies were separated from the other insects. The head and genitalia of each specimen were dissected and individually slide-mounted. The slide-mounting was done in Swan solution (chloral hydrate/acetic acid/Arabic gum). Entomological keys were used for species identification [9, 10]. The morphological identification of the species was based on specific features of the pharynx and external genitalia (males), and pharynx and internal genitalia (females). The morphological description of the new species was performed according to the available guideline [11]. The rest of the sand fly bodies were individually stored in 70% ethanol for molecular identification.

DNA was extracted individually from the thorax of 10 randomly selected specimens, five males and five females, morphologically identified as *Ph*. *neglectus*, and of a *Ph.* (*Transphlebotomus*) sp. female using the Qiagen DNeasy Blood and Tissue Kit (Qiagen, Austin, TX, USA), following the manufacturer’s instructions, and stored at −20 °C. Polymerase chain reaction (PCR) amplifications of the mitochondrial cytochrome c oxidase subunit 1 (*CO1*) gene region (~ 660 bp) were performed in 50 μl reaction volume using LCO1490 and HCO2198 primers [12]. Mitochondrial cytochrome b (*Cytb*) and NADH dehydrogenase subunit 4 (*ND4*) genes were also analysed for the *Ph.* (*Transphlebotomus*) sp. female. CB1/N1N-PDR and ND4C/ND4AR primer pairs were used for the amplification of the ~ 480 bp fragment of the *Cytb* and ~ 610 bp fragment of the *ND4* genes, respectively, as described earlier [13, 14]. The amplification products were separated and visualized on 2% agarose gels, purified using the QIAquick PCR Purification Kit (Qiagen), and directly sequenced in both directions using the primers used for DNA amplification (ABI Prism BigDye Terminator Cycle Sequencing Ready Reaction Kit, Foster City, CA, USA). Sequences were edited and aligned using BioEdit v.7.0.9.0 [15]. A BLAST search was conducted to compare all the obtained sequences with the ones deposited in the GenBank database. Maximum likelihood (ML) analysis of the obtained *Cytb* gene sequence and similar sequences available in GenBank was conducted in MEGA6.0 under the assumptions of a T92+G nucleotide substitution model [16]. For all the gene regions analysed, Kimura’s 2-parameter (K2P) genetic distances between the members of the subgenus *Transphlebotomus* were estimated. The TCS method implemented in PopART (Population Analysis with Reticulate Trees) [17] was used to construct haplotype networks.

## Results

### Sand fly morphological identification

**Fig. 2:**
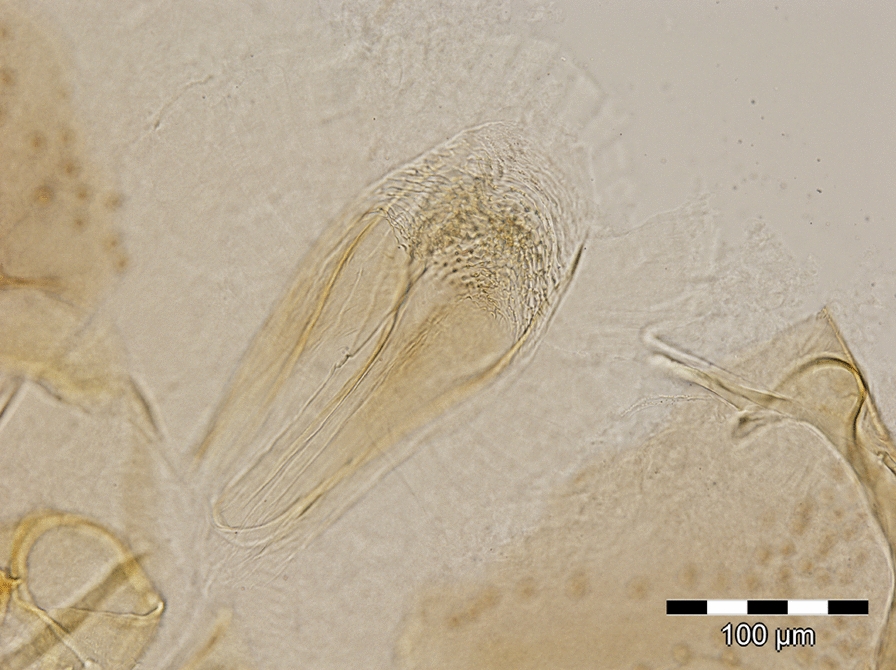
Morphological details of the pharynx for the female specimen of the *Phlebotomus simonahalepae* n. sp.

**Fig. 3:**
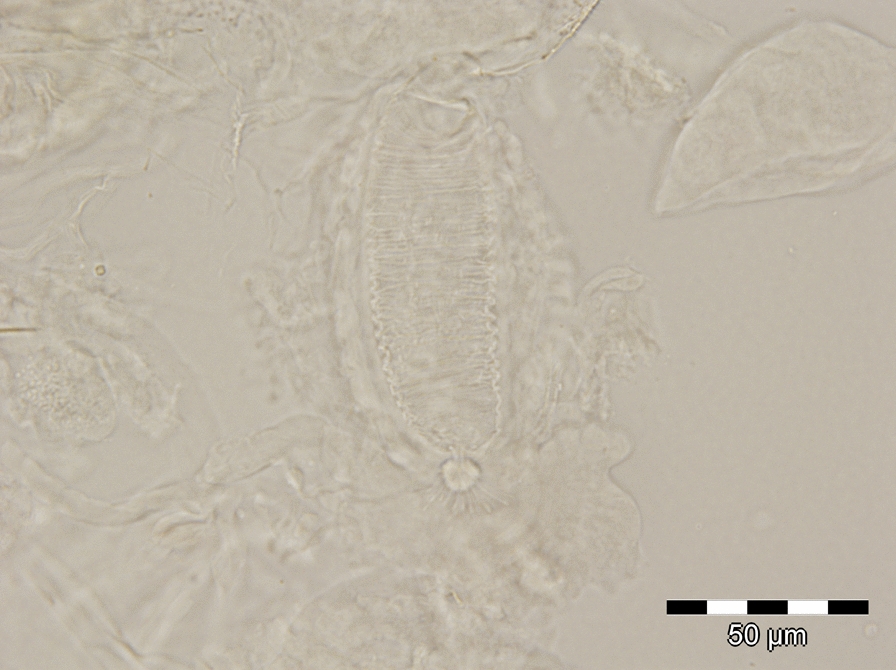
Morphological details of the spermathecae for the female specimen of the *Phlebotomus simonahalepae* n. sp.

A total of 645 sand flies were collected, of which 438 (67.9%) were females and 207 (32.1%) males. Six females were blood-fed (1.4%), and other five were gravid (1.1%) (Additional file 1: Table S1). In 2018, a total of 331 (94.3%) sand flies were recovered from the CDC light traps, and 20 (5.7%) were collected using mouth aspirators. In 2019, a total of 233 (79.3%) sand flies were recovered from the CDC light traps and 61 (20.7%) from sticky traps (Additional file 1: Table S1). All specimens belonged to genus *Phlebotomus*. A total of 644 (99.8%) specimens belonged to the subgenus *Larroussius* and were identified as *Ph*. *neglectus* (Additional file 1: Table S1). One female specimen (0.2%) was morphologically identified as a species of the subgenus *Transphlebotomus* based on the specific morphology of the pharynx (Fig. 2) and genitalia (Fig. 3). This female actually diverged morphologically and molecularly (see below) from known species of the subgenus *Transphlebotomus*.

### Molecular analyses

Amplification of the *CO1* gene region was successful for all the randomly selected *Ph*. *neglectus* specimens. Only one unique *CO1* haplotype was obtained for the 10 specimens analysed, and this haplotype showed 99.85% similarity with a *Ph*. *neglectus* specimen from Serbia (GenBank: KY848830).

**Fig. 4:**
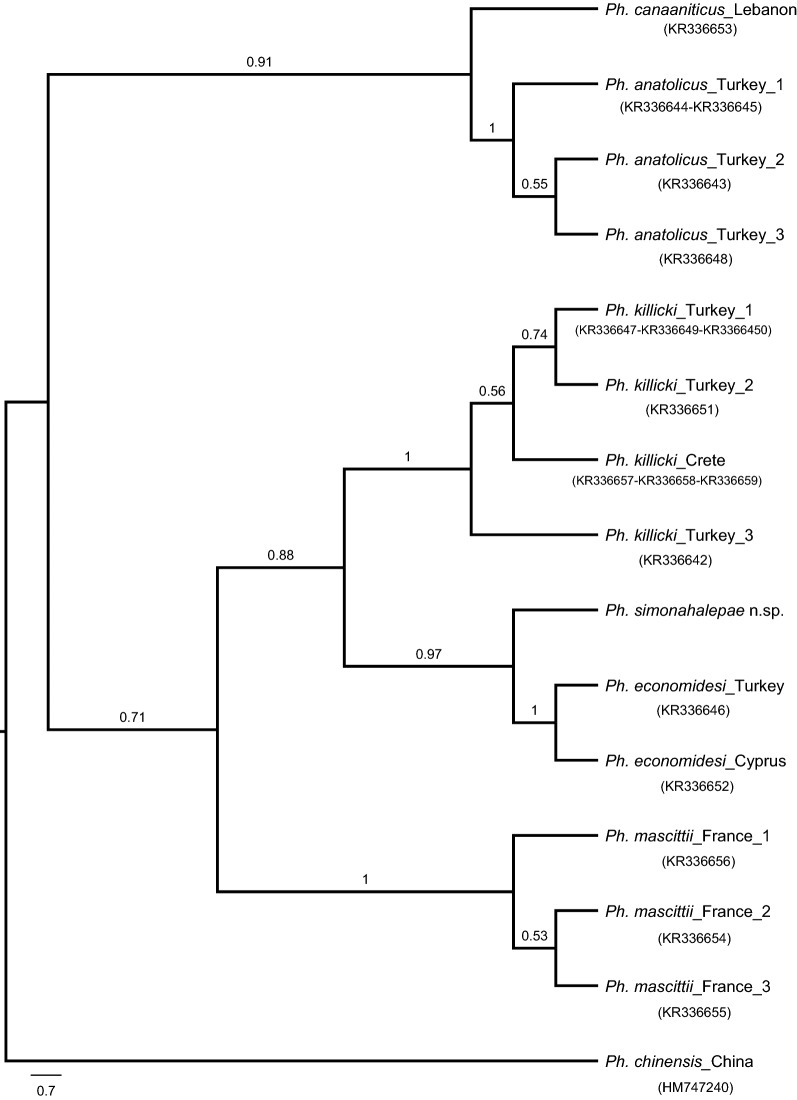
The ML tree with bootstrap values higher than 50% obtained for the members of *Transphlebotomus* subgenus. The sequences of *Ph*. *anatolicus*, *Ph*. *canaaniticus*, *Ph*. *economidesi*, *Ph*. *killicki*, *Ph*. *mascittii*, and *Ph*. *chinensis* were obtained from GenBank (KR336642-336659, HM747247)

The ML analysis of the *Cytb* haplotypes obtained previously for the other *Transphlebotomus* species together with the specimen from Romania revealed that this female was highly diverged from the rest of the formally described species within this subgenus. This specimen was placed as a sister taxon to *Ph*. *economidesi* from Cyprus (GenBank: KR336652) and Turkey (GenBank: KR336646) with a high genetic distance (7.5%) (Fig. 4). The *CO1* sequence divergence between the Romanian *Transphlebotomus* specimen and the rest of the members ranged from 9 to 14.6%. The *ND4* sequences available for the previously described *Transphlebotomus* species deeply diverged from the Romanian specimen (mean K2P = 7.5–11.6%) (Table 1). The female *Transphlebotomus* specimen was placed in an independent network for each of the gene regions analysed (Additional file 2: Figure S1).

**Table 1:** K2P sequence divergence (*Cytb*/*CO1*/*ND4*) between the species belonging to the subgenus *Transphlebotomus*

	*Ph*. *anatolicus*	*Ph*. *canaaniticus*	*Ph. economidesi*	*Ph*. *killicki*	*Ph*. *mascittii*	*Ph*. *simonahalepae* n. sp.
*Ph*. *anatolicus*						
*Ph*. *canaaniticus*	0.069/0.068/na					
*Ph. economidesi*	0.107/0.146/na	0.112/0.131/0.103				
*Ph*. *killicki*	0.107/0.146/na	0.103/0.111/0.097	0.083/0.131/0.096			
*Ph*. *mascittii*	0.107/0.146/na	0.120/0.104/0.116	0.116/0.150/0.105	0.106/0.115/0.117		
*Ph*. *simonahalepae* n. sp.	0.107/0.146/na	0.122/0.112/0.116	0.075/0.090/0.075	0.086/0.110/0.101	0.121/0.115/0.112	

*na* not available

**Family Psychodidae Newman, 1834**

**Genus** ***Phlebotomus*** **Rondani & Berté, 1840**

***Phlebotomus simonahalepae*** **Cazan, Erisoz Kasap & Mihalca, n. sp.**

***Type locality*** Canaraua Fetii Special Protection Area (44.07302 N, 27.64289 E), Dobrogea region, South-Eastern Romania.

***Type-material*** The holotype female (accession number 000528778100001) has been deposited in the ‘Grigore Antipa’ Natural History Museum, Bucharest, Romania.

***Representative DNA sequences*** GenBank accession numbers MZ647965 (*CO1*), MZ647523 (*Cytb*), MZ647524 (*ND4*).

***ZooBank registration*** To comply with the regulations set out in Article 8.5 of the amended 2012 version of the International Code of Zoological Nomenclature (ICZN) [18], details of the new species have been submitted to ZooBank. The Life Science Identifier (LSID) of the article is urn:lsid:zoobank.org:pub:524DD296-DD7C-401E-9576-8CAA8FCEAED1. The LSID for the new name *Phlebotomus simonahalepae* is urn:lsid:zoobank.org:act:DF49E8EC-7A8A-4A66-BBA5-058F82380E64.

***Etymology*** The species is dedicated to the famous tennis player Simona Halep, born in the same county as the type locality.

## Description

***Female*** [The counts and measurements provided below are those of the holotype (labelled RO-CAN62; museum record number: 000528778100001). The specimen was remounted for the second time due to a precipitation of the Swan solution between 2018 and 2020. In order to perform additional measurements, the authors have performed the second mounting].

**Fig. 5:**
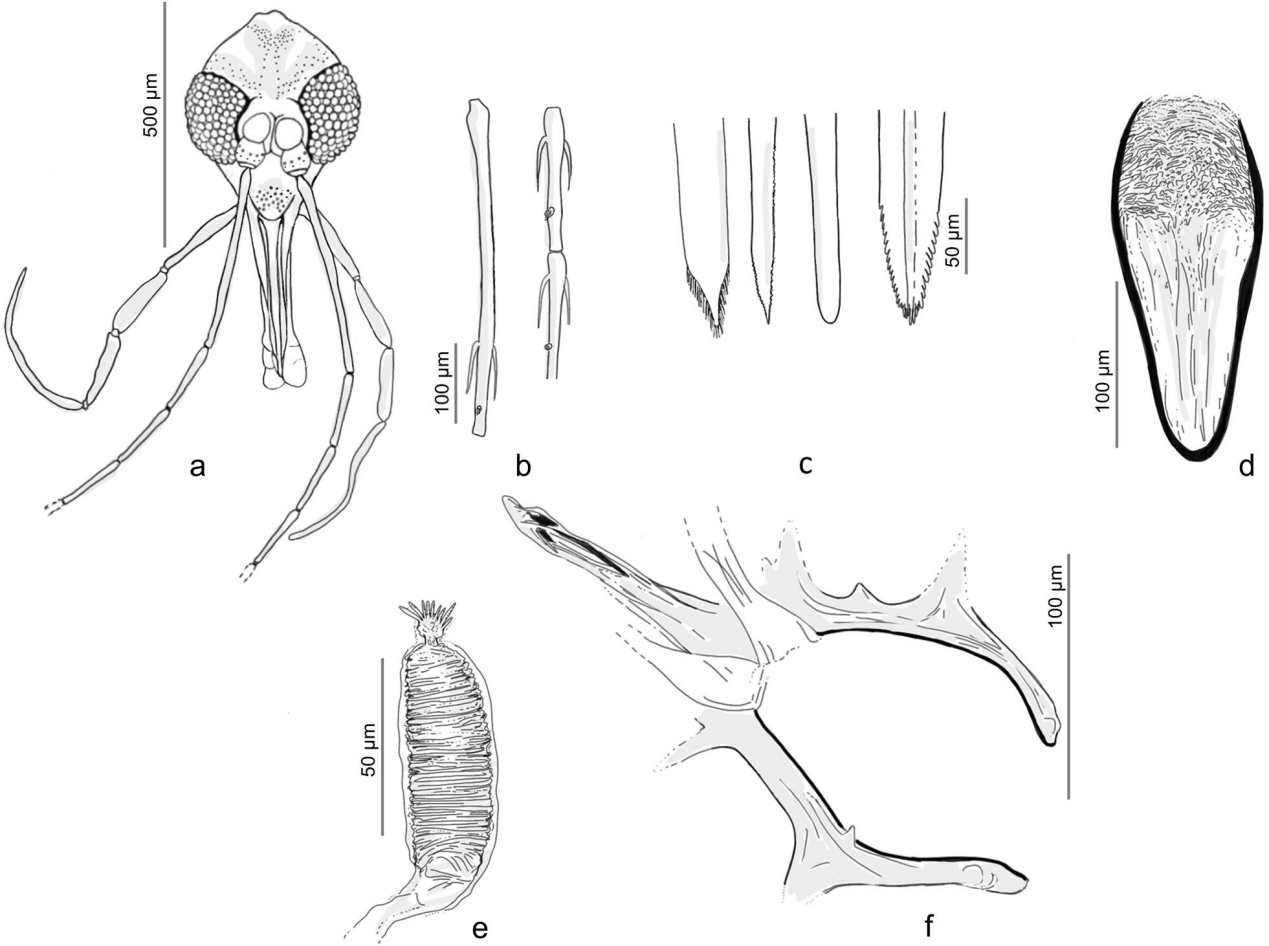
Female of *Phlebotomus simonahalepae* n. sp. Holotype. Included in the ML tree according to Fig. 4. **a** Head. **b** Flagellomeres 1, 2, 3. **c** Labrum—epipharynx, maxillary lacinia, mandible, hypopharynx (from left to right). **d** Pharynx. **e** Spermathecae. **f** Genital furca and spermathecae

*Head* (Fig. 5a). Occiput with two narrow lines of well individualised setae. On the line above the eyes, one greater insertion of seta on each side. Clypeus 192.95 μm long, 148.19 μm wide with 28 setae randomly distributed. Eyes 316.61 μm long, 248.15 μm wide with about 100 facets. Interantennal suture incomplete. Interocular sutures not reaching the interantennal one. Flagellomeres (Fig. 5b): f1 (495.64 μm) longer than f2 (197.96 μm) + f3 (194.52 μm); f12, f13, f14 were missing at the time of measurement, but were previously observed. Ascoidal formula: 2/f1–f14 with long ascoids, reaching the next article. Number of sensillae and simple setae per flagellomere are indicated in Table 2. Palpi (Fig. 5a): p1: 60.24 μm long; p2: 233.48 μm; p3: 223.53 μm; p4: 216.58 μm; p5: 490.46 μm. Palpal formula: 1, 4, 2, 3, 5. Only one Newstead’s sensillae visible in the middle of the third palpal segment, part of a larger group, but detached at the time of examination. No Newstead’s sensilla on other palpal segment. Presence of one spiniform seta on p3, six on p4, and 10 on p5. Labrum-epipharynx (Fig. 5c) 458.50 μm long. f1/*E* = 1.08. Maxillary lacinia (Fig. 5c) exhibiting 16 fine external and more than 40 fine internal teeth. Hypopharynx (Fig. 5c) with about 34 triangular teeth. Cibarium with fine lateral denticles observed. Pharyngeal armature (Fig. 5d) well developed, occupying the last third of the pharynx, made with small dots-like teeth and long triangular teeth. *Genitalia* (Fig. 5e, f). Spermathecae cylindrical in shape, length = 96.82 μm, width = 30.58 μm, striated and capsulated. Terminal knob (7.85 μm) round-shaped with nine finger-like prolongations (approx. 4–7 μm) connected by a thin neck (3.6 μm). Absence of common duct. Ducts not visible in the anterior part. The basal part wide and smooth. *Thorax*, *abdomen*, *wings*, *and legs*. Not observed.

**Table 2:** Ascoids, sensillae and simple setae number for flagellomeres on the holotype *Phlebotomus simonahalepae* n. sp.

Flagellomere	Number of ascoids	Number of sensillae	Number of simple setae
f1	2	1	10
f2	2	1	11
f3	2	1	19
f4	2		22
f5	2		31
f6	2		35
f7	2		28
f8	2		31
f9	2		31
f10–f14^a^	nd	nd	nd

^a^The last flagellomeres were missing at the time of examination

## Discussion

Prior to the description of *Ph*. *simonahalepae* n. sp., the subgenus *Transphlebotomus* Artemiev, 1984 included five species: *Phlebotomus mascittii* Grassi, 1908; *Ph*. *canaaniticus* Adler and Theodor, 1931; *Ph*. *economidesi* Léger, Depaquit and Ferté, 2000; *Ph*. *anatolicus* Erisoz Kasap, Depaquit, Alten, 2015; and *Ph*. *killicki* Dvorak, Votypka, Volf, 2015 [10].

**Fig. 6:**
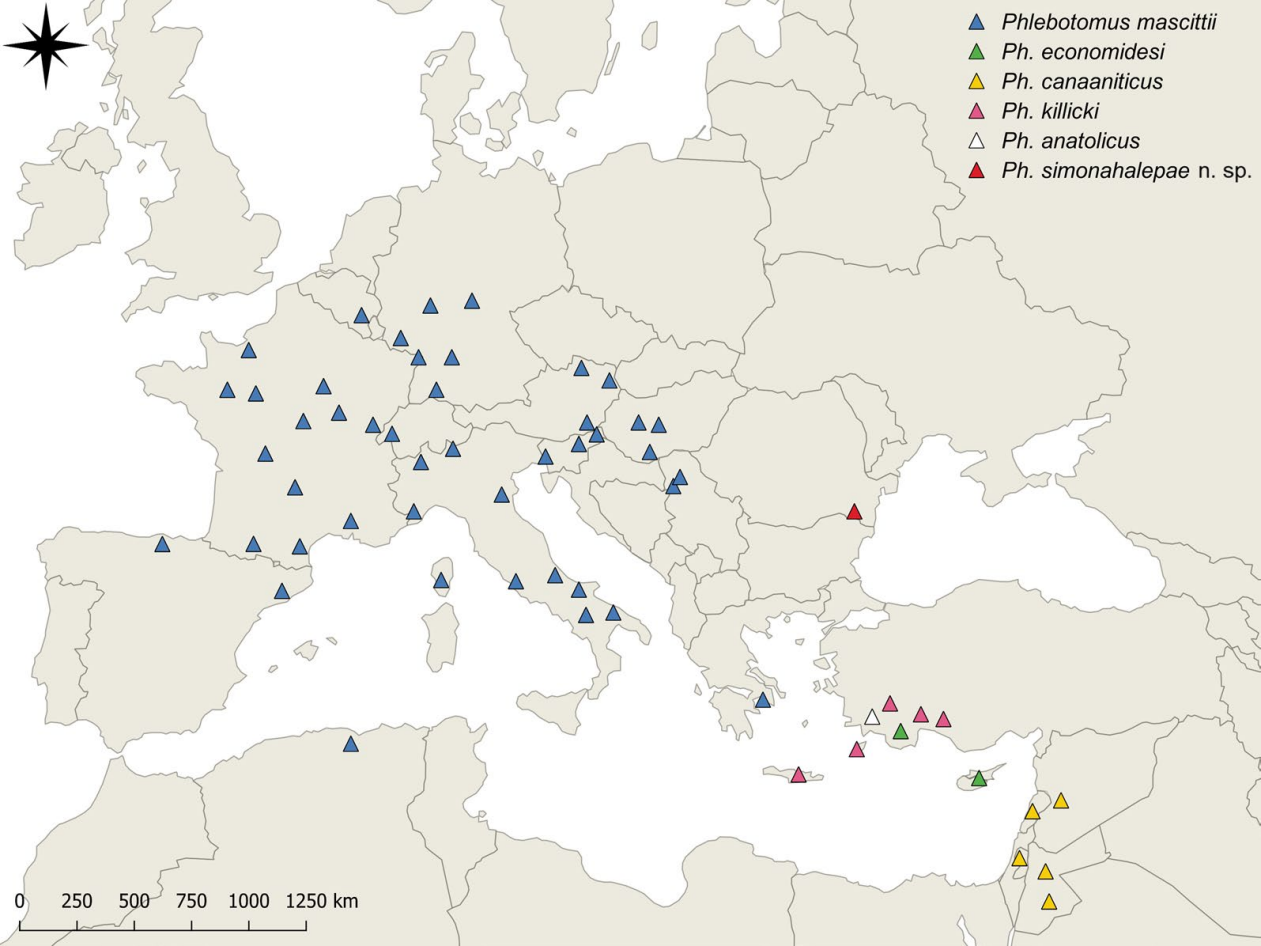
The currently known distribution areas of species of subgenus *Transphlebotomus* [19–30]

Considering the currently known distribution areas of species of subgenus *Transphlebotomus* (Fig. 6), it seems that *Ph*. *simonahalepae* n. sp. does not overlap with any of these species [19–30]. Despite repeated efforts to sample more specimens, no other individual from the newly described species was captured (authors’ unpublished data). For this reason, we were unable to examine more females and to describe the male of the new species.

When compared to females of other species of the subgenus, there are several morphological differences in *Ph*. *simonahalepae* n. sp., which together with the molecular analysis support the description of a new species. *Phlebotomus simonahalepae* n. sp. differs from *Ph*. *anatolicus* by the number of prolongations of the knob of the spermathecae (9 vs. 10–12), the presence of the neck as well as morphometry of the head structures [10]; from *Ph*. *mascittii* the morphological differences refer to the aspect of the knob of the spermathecae, number of prolongations (9 vs. 10–12), and the presence of the neck and the size of labrum [9]; from *Ph*. *canaaniticus* the morphological differences include the number of prolongations on the knob of the spermathecae (9 vs. 10–14) and size of labrum [9]; from *Ph*. *economidesi* the difference consists in the morphological aspect of the knob of the spermathecae and the presence of a thin short neck [31]. The morphological description of the female of *Ph*. *killicki* does not include details of the spermathecae to allow comparison. However, the morphometry of the head structures shows significant differences between *Ph*. *simonahalepae* n. sp. and *Ph*. *killicki* [10].

Besides the morphological differences, the description of the new species is also based on the analyses of the three mitochondrial DNA markers (*Cytb*, *CO1*, and *ND4* gene regions), which supported the monophyly of the subgenus *Transphlebotomus* and discriminated the five previously known species (*Ph*. *mascittii*, *Ph*. *canaaniticus*, *Ph*. *economidesi*, *Ph*. *anatolicus*, *Ph*. *killicki*) [10], as well as *Ph*. *simonahalepae* n. sp. Divergence of *Ph*. *simonahalepae* n. sp. from the rest of the *Transphlebotomus* species based on these three markers is comparable to those observed for several Old and New World sand fly species [32–34]. Congruently, independent haplotype networks obtained by parsimony analysis of these three data sets also suggest a new nominal species.

From a taxonomic point of view, the inclusion of *Ph*. *simonahalepae* n. sp. in the subgenus *Transphlebotomus* is justified by the morphology of the spermathecae, and its phylogenetic position obtained from molecular data [9].

Additionally, the data from this study revealed sand fly community composition changes since the last sampling in the area (Additional file 1: Table S1 and Table 3) [6]. *Phlebotomus neglectus* was the most abundant sand fly species recorded in the present study (99.8%), while in 1970 it was *Ph*. *balcanicus* and *Se*. *minuta* (Table 3). Both *Ph*. *neglectus* and *Ph*. *balcanicus* are vectors for *L*. *infantum*, but *Ph*. *neglectus* is the main one in south-central, southern, and eastern Europe, including Romania [2]. These changes could be explained by a series of factors, mainly environmental, demographic, and human behavioural factors, including the widespread use of insecticides in Romania during the malaria eradication programs (1958–1964) [5], the alterations of the sand fly habitats, or climate changes in the last decades [2], but also the different trapping methods used. Other changes in the sand fly species composition have also been observed in recent studies conducted in Romania [3, 35].

**Table 3:** Literature trapping data and sand fly composition in Canaraua Fetii, Romania (1968–1970) [6]

Collection date	*Ph*. *sergenti*			*Ph*. *balcanicus*			*Ph*. *neglectus*			*Se*. *minuta*			Total sand flies/collection date		
	T	M	F	T	M	F	T	M	F	T	M	F	T	M	F
27/06/1968	6	6	0	31	25	6	3	3	0	25	9	16	65	43	22
16/08/1968	1	0	1	4	4	0	11	10	1	26	9	17	42	23	19
27/09/1968	0	0	0	0	0	0	0	0	0	2	1	1	2	1	1
11/07/1969	3	0	1	15	12	3	10	9	1	0	0	0	28	23	5
26/06/1970	10	2	3	29	28	1	4	4	0	9	5	4	52	44	8
21/08/1970	7	7	0	3	3	0	11	9	2	20	13	7	41	32	9
30/09/1970	3	2	1	0	0	0	0	0	0	0	0	0	3	2	1
Total (1968–1970)	30	24	6	82	72	10	39	35	4	82	37	45	233	168	65

*T* total number of collected sand flies, *M* males, *F* females

## Conclusions

In the present study, the dominant sand fly species trapped in the Canaraua Fetii Protected Area (South-Eastern Romania) was represented by *Ph*. *neglectus*. One specimen was morphologically identified as belonging to the subgenus *Transphlebotomus*, and coupled morphological and molecular analysis led to the description of a new species, namely, *Ph*. (*Transphlebotomus*) *simonahalepae* n. sp.

## Supplementary Information

**Additional file 1: Table S1.** Trapping data included in the study with the recorded sand fly composition, Canaraua Fetii, Dobrogea Region.

**Additional file 2: Figure S1.** Haplotype networks obtained for the members of *Transphlebotomus* subgenus (**a** *Cytb*; **b** *CO1*; **c** *ND4*). The sequences of *Ph*. *anatolicus*, *Ph*. *canaaniticus*, *Ph*. *economidesi*, *Ph*. *killicki*, and *Ph*. *mascittii* were obtained from GenBank (AY780350, KF483664, KR336642-336659, KX869078, KX963380, KY848831, MN003381, MN812827-MN812830, MT332686–MT332688). Haplotypes are sized according to their relative frequencies. Different colors represent different species and black-filled circles represent missing haplotypes. The number of mutational steps are indicated by the dashes.

## Data Availability

All data generated or analysed during this study are included in this published article and its additional files. Sequences generated in this study are available in GenBank (MZ647965, MZ647523, MZ647524).

## Abbreviations

VL: Visceral leishmaniasis
CanL: Canine leishmaniasis
PCR: Polymerase chain reaction amplification
*CO1*: Mitochondrial cytochrome c oxidase subunit 1 gene region
*Cytb*: Mitochondrial cytochrome b gene
*ND4*: NADH dehydrogenase subunit 4 gene
ML: Maximum likelihood
K2P: Kimura’s 2-parameter genetic distances
