# Supplementary material for: Sand fly fauna of South-Eastern Romania, with the description of Phlebotomus (Transphlebotomus) simonahalepae n. sp. (Diptera: Psychodidae)
Source: Parasit Vectors. 2021 Sep 6;14:448. doi: 10.1186/s13071-021-04929-6 (PMC8420062; doi:10.1186/s13071-021-04929-6)
Supplement: Supplementary file 1 — Additional file 1: Table S1. Trapping data included in the study with the recorded sand fly composition, Canaraua Fetii, Dobrogea Region. [file 13071_2021_4929_MOESM1_ESM.docx]

| Collection date | Meteorological data | | | | Trap type | Trap set | Environmental details | *Ph*. *neglectus* | | | | *Ph*. (*Transphlebotomus*) sp. | | | | Total |
| --- | --- | --- | --- | --- | --- | --- | --- | --- | --- | --- | --- | --- | --- | --- | --- | --- |
|  | T min | T max | % RH | PP |  |  |  | M | F | G | BF | M | F | G | BF |  |
| 31/07/18 | 19 | 33 | 61 | 0.8 | Trap 1 (CDC) | Indoor | Ruins/building, traps set near the walls. | 3 | 12 | 0 | 0 | 0 | 0 | 0 | 0 | 15 |
| 31/07/18 | 19 | 33 | 61 | 0.8 | Trap 2 (CDC) | Indoor | Ruins/building, traps set near the walls. | 0 | 4 | 0 | 0 | 0 | 0 | 0 | 0 | 4 |
| 31/07/18 | 19 | 33 | 61 | 0.8 | Trap 3 (CDC) | Indoor | Ruins/building, traps set near the walls. | 0 | 0 | 0 | 0 | 0 | 0 | 0 | 0 | 0 |
| 01/08/18 | 18 | 32 | 60 | 0.4 | Trap 1 (CDC) | Indoor | Ruins/building, traps set near the walls. | 2 | 13 | 0 | 0 | 0 | 0 | 0 | 0 | 15 |
| 01/08/18 | 18 | 32 | 60 | 0.4 | Trap 2 (CDC) | Outdoor | Traps set a cave entrance, no rocks, just soil. | 0 | 0 | 0 | 0 | 0 | 0 | 0 | 0 | 0 |
| 01/08/18 | 18 | 32 | 60 | 0.4 | Trap 3 (CDC) | Outdoor | Traps set a cave entrance, no rocks, just soil. | 0 | 0 | 0 | 0 | 0 | 0 | 0 | 0 | 0 |
| 02/08/18 | 22 | 33 | 57 | 0 | Mouth Aspirator | Outdoor | Sand flies collected directly from the stone walls, at cave entrance | 10 | 9 | 0 | 0 | 0 | 0 | 0 | 0 | 19 |
| 02/08/18 | 22 | 33 | 57 | 0 | Mouth Aspirator | Outdoor | Sand fly collected while blood-feeding on a member of the team. | 0 | 1 | 0 | 1 | 0 | 0 | 0 | 0 | 1 |
| 02/08/18 | 22 | 33 | 57 | 0 | Trap 1 (CDC) | Outdoor | Traps set a rocky cave entrance. | 55 | 98 | 1 | 2 | 0 | 0 | 0 | 0 | 153 |
| 02/08/18 | 22 | 33 | 57 | 0 | Trap 2 (CDC) | Outdoor | Traps set a rocky cave entrance. | 14 | 29 | 0 | 0 | 0 | 1 | 0 | 0 | 44 |
| 02/08/18 | 22 | 33 | 57 | 0 | Trap 3 (CDC) | Outdoor | Traps set a rocky cave entrance. | 36 | 64 | 1 | 0 | 0 | 0 | 0 | 0 | 100 |
| 29/07/19 | 23 | 36 | 42 | 0 | ST | Outdoor | 20 sticky traps (148x 210 mm) set at cave entrance near rocky wall. | 21 | 5 | 0 | 0 | 0 | 0 | 0 | 0 | 26 |
| 29/07/19 | 23 | 36 | 42 | 0 | Trap 1 (CDC) | Indoors | Ruins/building, traps set near the walls. | 0 | 0 | 0 | 0 | 0 | 0 | 0 | 0 | 0 |
| 29/07/19 | 23 | 36 | 42 | 0 | Trap 2 (CDC) | Outdoor | Traps set a cave entrance, no rocks, just soil. | 0 | 0 | 0 | 0 | 0 | 0 | 0 | 0 | 0 |
| 29/07/19 | 23 | 36 | 42 | 0 | Trap 3 (CDC) | Outdoor | Traps set a rocky cave entrance. | 1 | 6 | 0 | 0 | 0 | 0 | 0 | 0 | 7 |
| 30/07/19 | 20 | 37 | 38 | 0 | ST | Outdoor | 20 sticky traps (148x 210 mm) set at cave entrance near rocky wall. | 11 | 5 | 1 | 1 | 0 | 0 | 0 | 0 | 16 |
| 30/07/19 | 20 | 37 | 38 | 0 | Trap 1 (CDC) | Indoors | Ruins/building, traps set near the walls. | 0 | 0 | 0 | 0 | 0 | 0 | 0 | 0 | 0 |
| 30/07/19 | 20 | 37 | 38 | 0 | Trap 2 (CDC) | Outdoor | Traps set a cave entrance, no rocks, just soil. | 0 | 0 | 0 | 0 | 0 | 0 | 0 | 0 | 0 |
| 30/07/19 | 20 | 37 | 38 | 0 | Trap 3 (CDC) | Outdoor | Traps set a rocky cave entrance. | 16 | 36 | 0 | 0 | 0 | 0 | 0 | 0 | 52 |
| 31/07/19 | 19 | 37 | 36 | 0 | ST | Outdoor | 20 sticky traps (148x 210 mm) set at cave entrance near rocky wall. | 1 | 7 | 0 | 0 | 0 | 0 | 0 | 0 | 8 |
| 31/07/19 | 19 | 37 | 36 | 0 | Trap 1 (CDC) | Indoors | Ruins/building, traps set near the walls. | 0 | 0 | 0 | 0 | 0 | 0 | 0 | 0 | 0 |
| 31/07/19 | 19 | 37 | 36 | 0 | Trap 2 (CDC) | Outdoor | Traps set a cave entrance, no rocks, just soil. | 0 | 0 | 0 | 0 | 0 | 0 | 0 | 0 | 0 |
| 31/07/19 | 19 | 37 | 36 | 0 | Trap 3 (CDC) | Outdoor | Traps set a rocky cave entrance. | 3 | 19 | 0 | 2 | 0 | 0 | 0 | 0 | 22 |
| 01/08/19 | 19 | 39 | 48 | 0 | ST | Outdoor | 20 sticky traps (148x 210 mm) set at cave entrance near rocky wall. | 2 | 9 | 1 | 0 | 0 | 0 | 0 | 0 | 11 |
| 01/08/19 | 19 | 39 | 48 | 0 | Trap 1 (CDC) | Indoors | Ruins/building, traps set near the walls. | 0 | 0 | 0 | 0 | 0 | 0 | 0 | 0 | 0 |
| 01/08/19 | 19 | 39 | 48 | 0 | Trap 2 (CDC) | Outdoor | Traps set a cave entrance, no rocks, just soil. | 0 | 0 | 0 | 0 | 0 | 0 | 0 | 0 | 0 |
| 01/08/19 | 19 | 39 | 48 | 0 | Trap 3 (CDC) | Outdoor | Traps set a rocky cave entrance. | 32 | 120 | 1 | 0 | 0 | 0 | 0 | 0 | 152 |

T min: minimum temperature; T max: maximum temperature; %RH: % relative humidity; PP: precipitations; CDC: miniatural CDC light traps; ST: sticky traps; F: female; M: male; G: gravid female; BF: blood fed female;
