# Supplementary figures and images for: Sand fly fauna of South-Eastern Romania, with the description of Phlebotomus (Transphlebotomus) simonahalepae n. sp. (Diptera: Psychodidae)
Source: Parasit Vectors. 2021 Sep 6;14:448. doi: 10.1186/s13071-021-04929-6 (PMC8420062; doi:10.1186/s13071-021-04929-6)

a

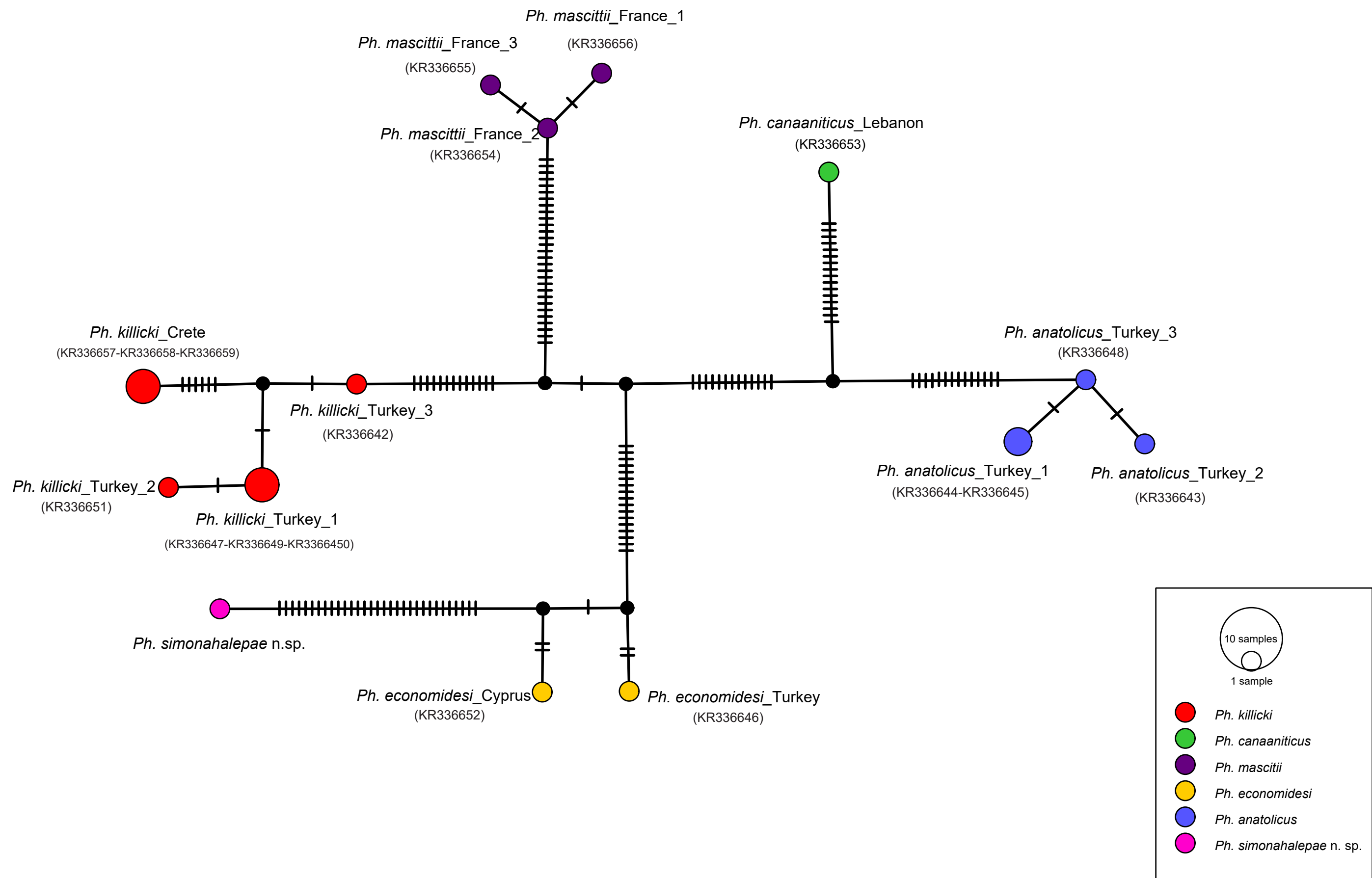

b

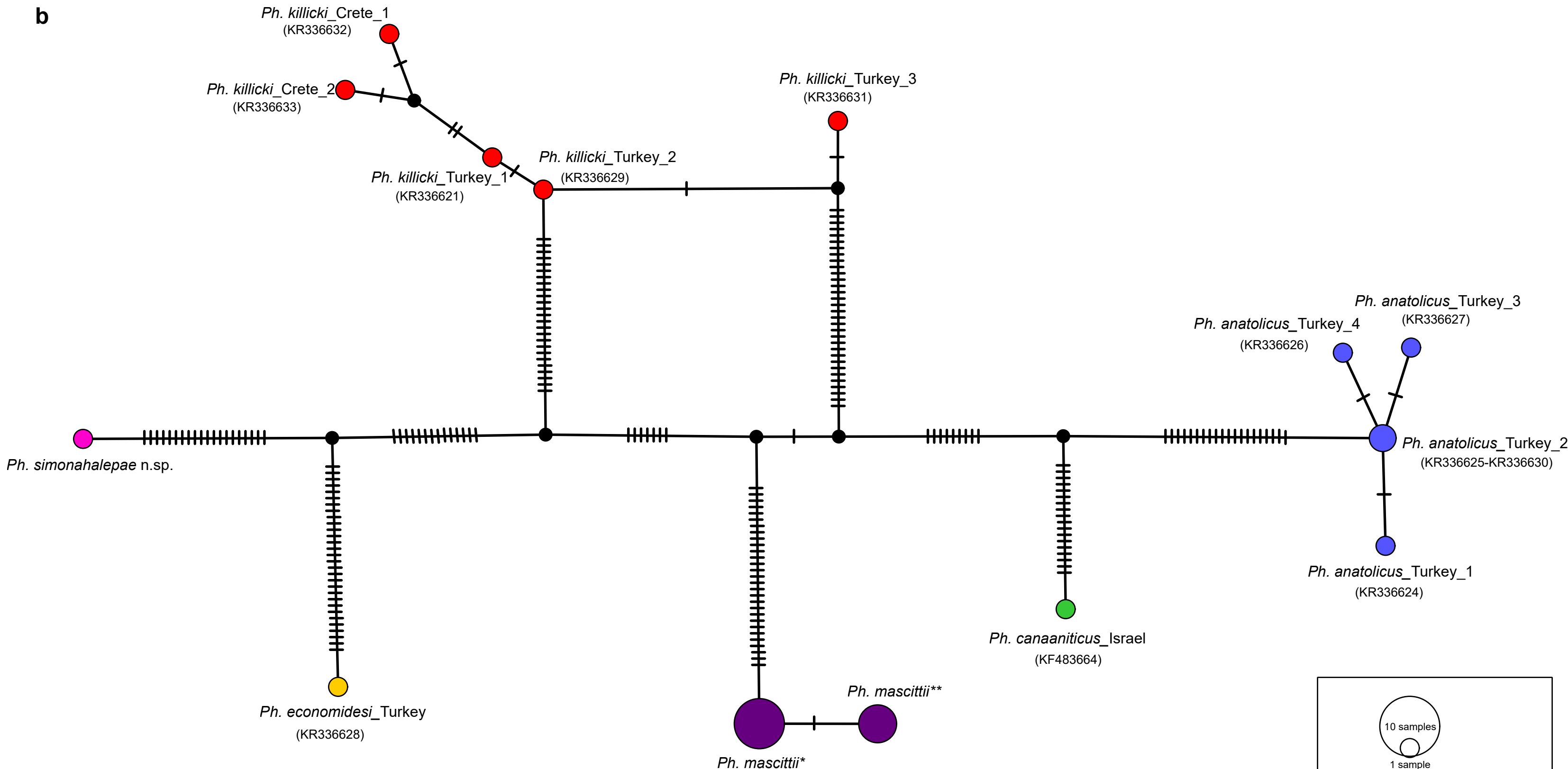

GenBank Accession Numbers: \* MN003381, MN312827, MN312828, MN312830  
MT332688, KX869078, KX981913  
\*\* MN812829, MT332686, MT332687, KX963380  
KY848831

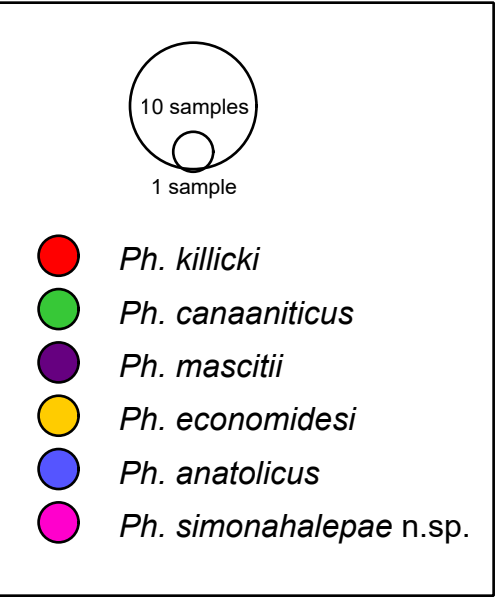

C

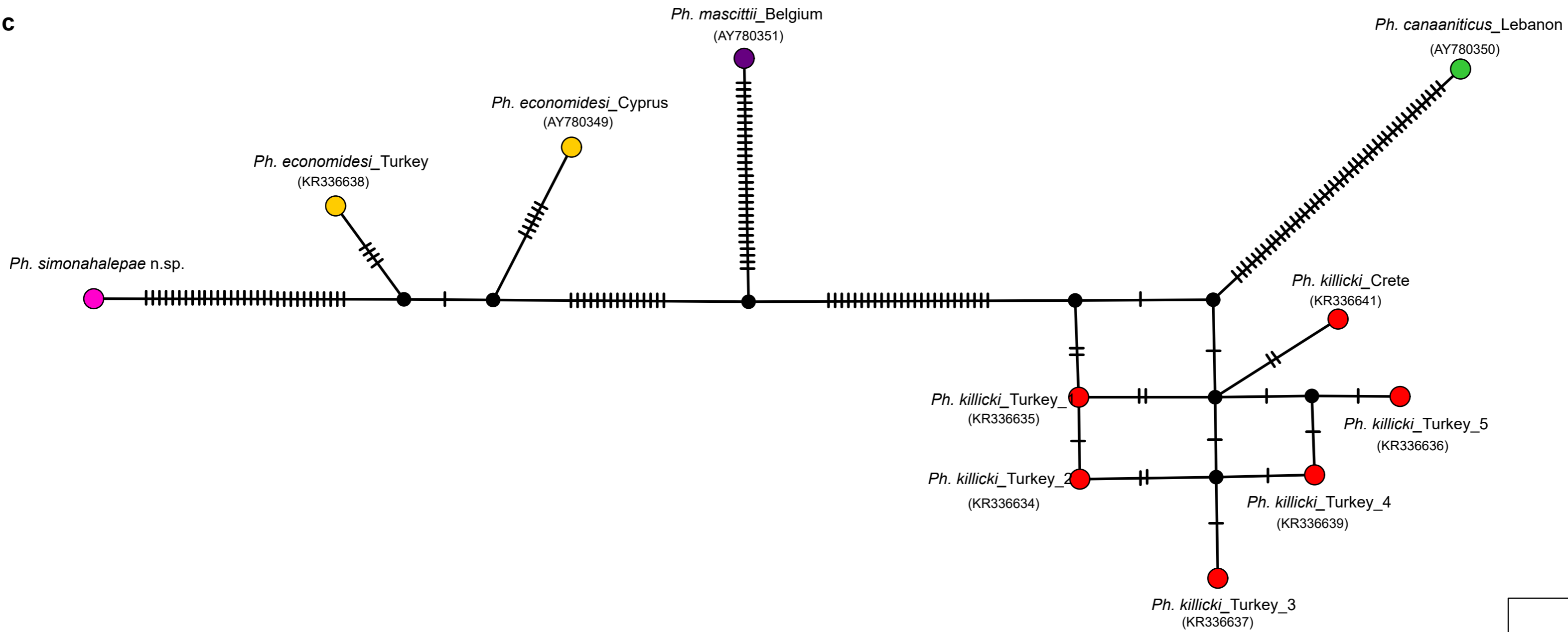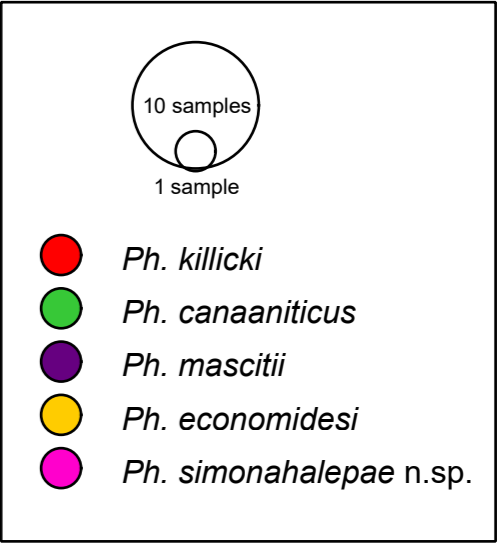

Supplement: Supplementary file 2 — Additional file 2: Figure S1. Haplotype networks obtained for the members of Transphlebotomus subgenus (a Cytb; b CO1; c ND4). The sequences of Ph. anatolicus, Ph. canaaniticus, Ph. economidesi, Ph. killicki, and Ph. mascittii were obtained from GenBank (AY780350, KF483664, KR336642-336659, KX869078, KX963380, KY848831, MN003381, MN812827-MN812830, MT332686–MT332688). Haplotypes are sized according to their relative frequencies. Different colors represent different species and black-filled circles represent missing haplotypes. The number of mutational steps are indicated by the dashes. [file 13071_2021_4929_MOESM2_ESM.pdf]
